# Neurophysiological Signatures of Major Depressive Disorder and Frontocentral Gamma Auditory Response Deficits

**DOI:** 10.1155/da/7390951

**Published:** 2025-02-19

**Authors:** Xiaoya Liu, Shuang Liu, Fangyue Su, Wenquan Zhang, Yufeng Ke, Dong Ming

**Affiliations:** ^1^Medical School of Tianjin University, Tianjin University, Tianjin, China; ^2^College of Precision Instruments and Optoelectronics Engineering, Tianjin University, Tianjin, China

## Abstract

**Background:** Aberrant gamma oscillations in major depressive disorder (MDD) have attracted extensive attention, but evidence delineating such neural signatures is lacking. The auditory steady-state response (ASSR) elicited by periodic auditory stimuli is a robust probe of gamma oscillations. Here, we sought to characterize early transient auditory evoked responses (AEPs) and sustained gamma ASSRs in MDD, thereby identifying reliable neurophysiological signatures and providing preliminary interpretations of gamma auditory response deficits in MDD.

**Methods:** Electroencephalographic data were obtained from 40 first-episode drug-naïve patients with MDD and 41 demographically matched healthy controls (HCs) during a 40-Hz ASSR paradigm, encompassing two periodic stimuli—chirp and click stimuli. Source analysis of transient AEPs was performed to identify generators involved in early information processing dysfunction. In addition, spectrotemporal and spatial characteristics of 40-Hz ASSRs were analyzed using event-related spectral perturbation, inter-trial phase coherence, and functional connectivity index.

**Results:** Compared to HCs, patients showed a reduced P200 amplitude that was source-localized to the middle temporal gyrus, possibly reflecting an underlying impairment in the processes of early allocation or auditory information perception within the auditory pathways. Meanwhile, attenuated 40-Hz power and phase coherence, in conjunction with suppressed right frontotemporal and frontocentral connectivity, were observed in MDD, highlighting the multidimensional entrained gamma inhibition. Correlation analyses revealed that the decreased right frontocentral connectivity was strongly related to increased anxiety severity. Importantly, these abnormalities correlated with the patient's symptoms were only found with the chirp stimulus, suggesting that the chirp stimulus has tremendous potential to reveal specific neurophysiological signatures of MDD.

**Conclusions:** Our data reveal impaired gamma auditory responses in first-episode drug-naïve patients with MDD and suggest that right frontocentral connectivity elicited by the chirp stimulus may represent a promising signature for predicting clinical symptoms.

## 1. Introduction

Major depressive disorder (MDD) is a serious, debilitating psychiatric disorder characterized by persistent anhedonia, feelings of worthlessness, and cognitive dysfunction [1]. It is a leading cause of disability worldwide and increases the risk of suicide [2]. However, the diagnosis of MDD remains entirely based on subjective symptomatology, with a certain degree of concealment. This may partly account for the high misdiagnosis rate of MDD [3, 4], necessitating the identification of neurobiological signatures to advance the mechanistic understanding of depressive psychopathology and ultimately yield important clinical implications.

Accumulating evidence over the past decades has suggested that gamma oscillations may be a predictor of MDD [5–7]. The role of gamma-aminobutyric acid-containing (GABAergic) inhibitory interneurons and excitatory pyramidal neurons in the generation of gamma oscillations has been extensively confirmed by neuroimaging data [8], and the abnormalities in GABAergic interneurons have been repeatedly reported in MDD [8–13]. In our previous electroencephalography (EEG) studies, we found a marked alteration of spontaneous gamma activity in first-episode drug-naïve patients with MDD, which was strongly correlated with depression severity [14, 15]. However, gamma oscillations are typically neglected due to their low amplitude properties and possible physiological artifacts [16, 17]. A common approach to enhance gamma activity is entrainment by exogenous rhythmic stimulation. The auditory steady-state response (ASSR), a fruitful probe of gamma synchronous activity, is a cortical oscillation entrained to both the frequency and phase of periodic auditory stimuli [18, 19]. In humans, the ASSR has been found to be remarkably pronounced at 40 Hz [20].

Although there have been some pathologic investigations of 40-Hz ASSRs in psychosis [21, 22], they provide little insight into MDD. To date, only two prospective studies have examined 40-Hz ASSRs in MDD [23, 24]. Most importantly, the evidence for 40-Hz ASSRs in MDD is derived from patients on pharmacological treatment. Pharmacological interventions have been shown to increase gamma power [25], thus explaining the absence of profound 40-Hz ASSR deficits in MDD, possibly due to auditory responses that cannot be disentangled from medication effects [23]. To rule out confounding medication effects, we examined 40-Hz ASSRs in a group of first-episode drug-naïve patients with MDD in the present study. In addition, previous studies of MDD have assessed 40-Hz ASSRs separately, either by power changes or phase abnormalities. However, changes in brain connectivity patterns of gamma auditory responses have not been investigated. Recent evidences suggest that the connections between brain regions support the latent mechanisms of cognitive function [26]. Therefore, mapping the connectivity patterns of 40-Hz ASSRs between spatially distributed brain regions may contribute to the elucidation of the pathophysiological mechanisms underlying auditory dysfunction in MDD.

Typically, auditory responses to repetitive auditory stimuli at 40 Hz can be decomposed not only into 40-Hz ASSRs but also into transient auditory evoked responses (AEPs; e.g., N100 and P200) [27–29]. Disturbances in AEPs elicited by the auditory oddball paradigm have been documented in MDD [30–32]. The characteristics of AEPs are known to represent a distinct pattern of cognitive mechanisms. Specifically, the N100 component reflects early information extraction during stimulus perception [33], localized in the primary auditory cortex and adjacent belt regions of the secondary auditory cortex [34, 35]. The P200 component reflects allocation processes [36], which may reflect overlapping activity in both temporal and frontal cortices [37]. Combined analysis of AEPs and 40-Hz ASSRs may provide a comprehensive understanding of neural circuit abnormalities in auditory information processing in MDD. Furthermore, enhancement of AEPs and 40-Hz ASSRs can be achieved by optimizing stimulus parameters—such as stimulus type [38, 39]. Chirp stimulus, which was developed to compensate for the cochlear traveling wave delay [40], is considered to be more sensitive in capturing gamma oscillations, mainly because of the higher signal-to-noise ratio [37, 41–44].

Here, we sought to characterize the transient AEPs and sustained 40-Hz ASSRs in first-episode drug-naïve patients with MDD, thereby identifying a neurophysiological signature and providing preliminary insight into the neural underpinnings of gamma auditory response deficits. We compared the N100 and P200 components in combination with source analysis to clarify the primary generators of early AEP changes. Moreover, event-related spectral perturbation (ERSP), inter-trial phase coherence (ITC), and weighted phase lag index were extracted to identify the spectrotemporal and spatial characteristics of 40-Hz ASSRs. Correlation analyses were performed to elucidate whether the altered EEG signatures could predict clinical symptom severity. In addition, based on our previous work [43], we further hypothesized that MDD-related differences in gamma auditory responses would be more pronounced with the chirp stimulus, ultimately providing a clinical avenue to explore the pathophysiological signatures of MDD.

## 2. Materials and Methods

### 2.1. Participants

First-episode drug-naïve patients with MDD (*n* = 40) and healthy controls (HCs, *n* = 41) were recruited from Tianjin Anding Hospital. All participants in both groups had normal or corrected-to-normal hearing and were matched for age, sex, educational level, handedness, and ethnicity (Table 1). Approval was obtained from the Ethics Committee of Tianjin Anding Hospital, and written informed consent was obtained from all participants.

The Structured Clinical Interview for the Diagnostic and Statistical Manual for Mental Disorders (DSM)-V [45] confirmed the diagnosis of MDD, and a score of ≥14 on the 17-item Hamilton Rating Scale for Depression (HAMD-17) [46] and a score of ≤10 on the Young Mania Rating Scale (YMRS) [47] confirmed an active depressive episode. MDD is often associated with higher levels of anxiety; therefore, the Hamilton Anxiety Scale (HAMA) [48] was used to assess anxiety levels (Table 1). Exclusion criteria were (i) previous or current organic brain disorders or neurological disorders; (ii) previous or current exposure to electroconvulsive therapy or psychotropic treatments; and (iii) history of alcohol and drug abuse. HCs were excluded if they or their first-degree relatives had a self-reported psychiatric disorder.

### 2.2. Stimuli and Paradigm

**Figure 1:**
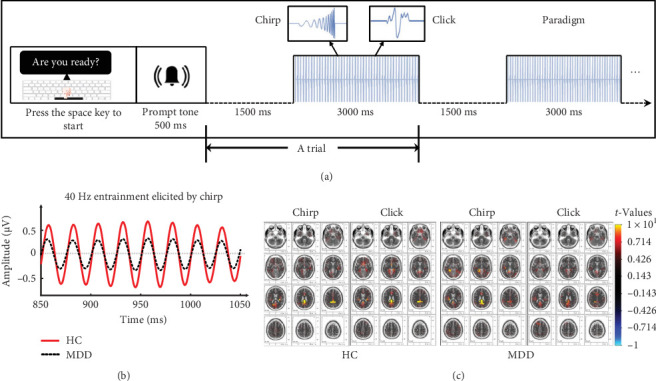
The ASSR paradigm and brain regions showing stimulus-elicited 40-Hz responses. (a) The task flow and the waveforms of the two signals (chirp and click). (b) An example of whole-brain averaged 40-Hz narrowband filtered signals from MDD (black dashed line) and HC (red solid line) groups elicited by the chirp stimulus. Data show clear entrainment with stimulation frequency in both groups. (c) Source localization of 40-Hz entrainment responses. For the HC group (left), significant 40-Hz activity was localized in the cingulate gyrus during both stimuli. For the MDD group (right), significant 40-Hz activity was localized separately in the posterior cingulate during the chirp stimulus and in the subgyral during the click stimulus. Red voxels indicate areas of relative activation (500–2500 ms auditory stimulus duration vs. 500-ms baseline). ASSR, auditory steady-state response; HC, healthy control; MDD, major depressive disorder.

Two stimulus types (i.e., chirp and click) were applied in the ASSR paradigm. The chirp stimulus [49, 50] was generated according to the following formula:

(1) $$\begin{array}{c} s\left(t\right)=A\left(t\right)\sin\left(\varphi \left(t\right)-\varphi_{0}\right), \end{array}$$

where *φ*_0_ determines the starting phase of the chirp signals; the phase *φ*(*t*) and amplitude factor *A*(*t*) were calculated as follows:

(2) $$\begin{array}{c} \varphi \left(t\right)=2\pi c^{2}\left(\frac{1}{t_{0}-t}-\frac{1}{t_{0}}\right), \end{array}$$

(3) $$\begin{array}{c} A\left(t\right)=\sqrt{\frac{2c^{2}}{{\left(t_{0}-t\right)}^{3}}}, \end{array}$$

(4) $$\begin{array}{c} t_{0}=cf^{\alpha }, \end{array}$$

where *f* = 50 Hz, the constants *c* = 0.15, and *α* = −0.5. The click stimulus consisted of 1-ms bursts of white noise, similar to that used previously [21, 23, 43, 51]. Both stimuli were presented using MATLAB 2020a (The MathWorks. Inc., Natick, MA, USA) with the Psychtoolbox [52]. The typical session for the click or chirp stimulus is shown in Figure 1a, each consisting of 28 trials. The duration of each trial was 3000 ms with an inter-trial interval of 1500 ms [27]. Auditory stimuli were presented binaurally at 40 Hz with a sampling rate of 44,100 Hz; the sound pressure level was set at 45 dB. Participants were asked to minimize their movements, stay awake, keep their gaze on the screen directly in front of them, and not pay attention to the stimuli in the paradigm. Specifically, the order of the click and chirp sessions was randomized across participants.

### 2.3. EEG Acquisition and Preprocessing

EEG was acquired using SynAmps RT amplifiers (NeuroScan Inc., Herndon, VA, USA) with 64 scalp electrodes arranged according to the international 10/20 system. All electrodes were referenced to the left mastoid electrode (“M1”) at a sampling rate of 1000 Hz, and the impedance of all electrodes was <10 kΩ.

EEG data were preprocessed using the EEGLAB toolbox [53] and custom MATLAB scripts. Imported data were rereferenced offline to the average bilateral mastoid electrodes ([“M1” + “M2”]/2) and low-pass filtered at 50 Hz. Subsequently, epochs were defined as nonoverlapping data segments from 1000 ms before stimulus onset to 3000 ms after stimulus onset and were discarded if the voltage at any electrode site exceeded 100 μV. Artifacts due to eye blinks, eye movements, and the heartbeat signal were removed using independent component analysis. The number of epochs after artifact removal from each group for each stimulus was as follows: HC (click: 26.59 ± 1.43, ≥23; chirp: 26.44 ± 1.60, ≥22) and MDD (click: 26.40 ± 0.93, ≥23; chirp: 26.08 ± 1.47, ≥21). A mixed model analysis of variance (ANOVA) with stimulus (chirp, click) as a within-subjects factor and group (MDD, HC) as a between-subjects factor, revealed no significant main effects (group: *F*_(1,79)_ = 0.975, *p* = 0.326; Stimulus: *F*_(1,79)_ = 3.233, *p* = 0.076) or interactions (*F*_(1,79)_ = 0.465, *p* = 0.497) for the remaining number of epochs. Figure 1b demonstrates the whole-brain averaged 40-Hz waveform elicited by the chirp stimulus, showing that clear stimulus frequency entrainment was evoked in both groups.

In addition, eight regions of interests (ROIs) were defined for inter-regional connectivity analyses, namely the left frontal (FL; FP1, AF3, F7, F5, F3, F1, FC5, FC3, and FC1), left temporal (TL; FT7, T7, and TP7), left central (CL; C5, C3, C1, CP5, CP3, and CP1), left parietal (PL; P7, P5, P3, P1, PO7, PO5, and PO3), right frontal (FR; FP2, AF4, F8, F6, F4, F2, FC6, FC4, and FC2), right temporal (TR; FT8, T8, and TP8), right central (CR; C6, C4, C2, CP6, CP4, and CP2), and right parietal (PR; P8, P6, P4, P2, PO8, PO6, and PO4) regions.

### 2.4. Event-Related Potential Analysis

Transient AEPs were assessed in all participants. In analogy to previous AEP studies [54, 55], we focused on central electrode sites (FCZ, CZ, and CPZ) to explore group differences. Epochs were low-pass filtered at 30 Hz and baseline corrected (from –300 to –100 ms). The N100 and P200 were extracted in the poststimulus windows of 70–140 ms and 140–260 ms, respectively [32].

### 2.5. Time–Frequency Analysis

ERSP and ITC were calculated to determine the changes in gamma oscillations, using a short-time Fourier transform with a Gauss window size of 1023 points. ERSP and ITC in the frequency range from 1 to 50 Hz were mapped to obtain a general idea of the frequency distribution. Then, 40-Hz entrainment responses (frequency range: 32–48 Hz) were extracted independently for each group within a time frame of 1000–2500 ms poststimulus. All data were expressed as the relative change from the baseline period (–300 to –100 ms), and ERSP values were averaged across epochs. Notably, one of the patients was excluded due to outliers (exclusion criterion: ERSP measures exceeding 4 SDs from the mean).

### 2.6. Connectivity Analysis

Inter-regional connectivity was measured using the weighted phase-lag-index (WPLI) [56], which is a measure of the synchronization between the signal pair over time and has been shown to be insensitive to almost-zero- and zero-lagging synchronization between signals [57]. Its mathematical formulation is as follows:

(5) $$\begin{array}{c} \text{WPLI}=\left|\frac{\sum_{t=1}^{n}\left|\text{imag}\left(S_{xy,t}\right)\right|\text{sgn}\left(\text{imag}\left(S_{xy,t}\right)\right)}{\sum_{t=1}^{n}\left|\text{imag}\left(S_{xy,t}\right)\right|}\right|, \end{array}$$

where *S*_*xy*,*t*_ indicates the complex cross-spectral density of the time series *x*(*t*) and *y*(*t*) at the time point *t* and || denotes the absolute value. The WPLI ranged from zero (no phase synchronization) to one (perfect phase synchronization). Here, WPLI of each pair of electrodes within the 38–42 Hz range was calculated for each epoch, then averaged across epochs, resulting in a connectivity matrix per participant. Further analysis was performed at a regional level of spatial resolution. The regional WPLI was based on the eight ROIs. For each ROI, the average connectivity of electrodes overlying the corresponding regions to all the electrodes belonging to the other ROIs was determined. In addition, the connectivity between any two ROIs was calculated as the average of all the electrode pairs between the two ROIs.

### 2.7. Source Estimation

Source-level estimation of AEPs and 40-Hz ASSRs was performed using standardized low-resolution brain electromagnetic tomography (sLORETA) [58]. A total of 6239 voxels at 5-mm spatial resolution were registered. To control for multiple comparisons, all statistical tests were performed using the implemented statistical nonparametric mapping (SnPM) tool, derived from 5000 randomizations. Significant difference *t*-values were presented in Talairach space.

For 40-Hz ASSRs, a separate paired *t*-test was performed for each group during each stimulus by contrasting the auditory stimulus periods (500–2500 ms after stimulus onset) with the baseline (−500 to 0 ms) for 40-Hz entrainment responses. In HC, the cingulate gyrus (Brodmann areas [BAs] 31) was activated (click: *t* = 11.520, *XYZ* = –10, –45, 35; chirp: *t* = 12.738, *XYZ* = –20, –45, 25). In MDD, the activating areas included the subgyral (click: *t* = 9.616, *XYZ* = –20, –50, 35; BA 31) and posterior cingulate (chirp: *t* = 12.393, *XYZ* = –5, –45, 25; BA 23). It can be speculated that the limbic lobe (BA 23 and BA 31; Figure 1c) plays a key role in the sustained auditory processing of continuous repetitive gamma-band stimuli.

For AEPs, paired and independent samples *t*-tests of N100 and P200 were performed (i) to compare stimulus-specific N100 sources between chirp and click stimuli and (ii) to compare P200 sources between MDD and HC, separately for the two stimuli. To control for multiple comparisons, all statistical tests used a nonparametric test, derived from 5000 randomizations.

### 2.8. Statistical Analyses

Mann–Whitney *U*-tests were performed for age and education between the two groups, as they did not meet the parametric assumptions of normal distribution by the Kolmogorov–Smirnov test. Chi-squared tests were performed for sex, handedness, and ethnicity. Independent samples *t*-tests were used to compare HAMD-17 and HAMA scores between the two groups.

For the primary analyses of 40-Hz ERSP and ITC, electrodes with main group differences were evaluated using the nonparametric permutation (*n* = 5000) *t*-test. Permutation tests were performed using the MATLAB function “mult_comp_perm_t2” [59]. The *p*-value was adjusted using the “tmax” method for multiple comparisons to control for family-wise error [59]. The averaged ERSP and ITC from the electrodes covering the significantly different entrainment activity between the two groups were further extracted for secondary analyses.

Secondary analyses evaluated HC vs. MDD for each dependent signature (AEPs, ERSP, ITC, and WPLI). Mixed model ANOVAs were performed for the within-subjects factor of Stimulus (chirp, click) and the between-subjects factor of Group (MDD, HC). Greenhouse–Geisser correction was applied for sphericity violations and Bonferroni corrections were applied for the post hoc pairwise comparisons. In addition, two-tailed Spearman's correlation analysis was performed to explore the relationships between clinical symptoms and EEG signatures.

## 3. Results

### 3.1. Early Auditory Processing Deficits in MDD

**Figure 2:**
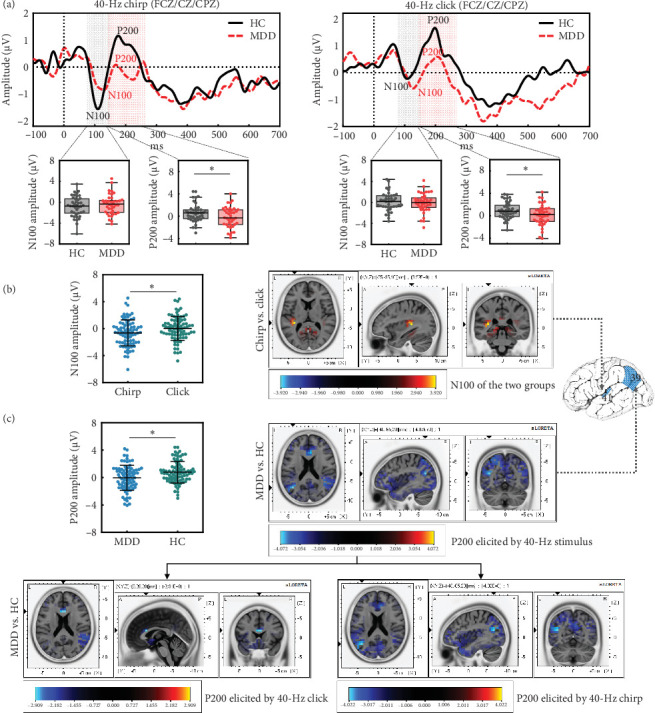
Transient AEPs during chirp (left) and click (right) stimuli. (a) The average AEP waveforms (top) at FCZ, CZ, and CPZ electrode sites from the MDD (red dashed line) and HC (black solid line) groups. Time 0 indicates stimulus onset. Box plots (bottom) with mean (white cross) and median (solid line) show N100 and P200 amplitudes separately for the HC (black dots) and MDD (red dots) groups. (b) Swarm plots of N100 amplitudes elicited by chirp (blue dots) and click (green dots) stimuli separately and the source localization of the N100 component (chirp vs. click) for all participants. A larger N100 activity was detected during the chirp stimulus and localized in the transverse temporal gyrus. (c) Swarm plots of P200 amplitudes between MDD (blue dots) and HC (green dots) groups and source localization of the P200 component (MDD vs. HC) for both stimuli. A significant reduction was detected in MDD, localized in the middle temporal gyrus (upper right), especially during the chirp stimulus (bottom right), but not during the click stimulus (bottom left). *⁣*^*∗*^*p* < 0.05. AEPs, auditory evoked responses; MDD, major depressive disorder.

The grand-averaged AEP waveforms elicited by the chirp and click stimuli were characterized by a negative N100 deflection in the range of 70–140 ms after stimulus onset, followed by a positive P200 deflection in the range of 140–260 ms (Figure 2a). A mixed model ANOVA revealed a significant main effect of Stimulus (*F*_(1, 79)_ = 6.523, *p* = 0.013; Figure 2b), as the chirp stimulus (mean ± standard error [*M* ± SE] = –0.616 ± 0.214) elicited a larger N100 compared to the click stimulus (*M* ± SE = 0.036 ± 0.198); however, the main effect of Group or the interaction effect of Group × Stimulus was not significant for the N100 component.

For the P200 component, a significant main effect of Group was observed (*F*_(1, 79)_ = 8.249, *p* = 0.005; Figure 2c), with MDD showing a reduced P200 amplitude (*M* ± SE = –0.037 ± 0.203) compared to HC (*M* ± SE = 0.781 ± 0.200). There were no Stimulus and Group × Stimulus interaction effects.

### 3.2. Source Reconstruction of AEP Components

Source localization analysis for the AEP components was performed only for the significant effects revealed by the scalp domain analyses, in order to find the generators responsible for the observed AEP changes. A voxel-by-voxel statistical analysis was performed by juxtaposing chirp vs. click conditions and MDD vs. HC groups, separately for N100 and P200. Tables 2 and 3 detail the spatial information of significant voxels for the N100 and P200 components. Figure 2b,c shows the LORETA *X*, *Y*, and *Z* slices around the maximum (*t*-score) condition and group differences, respectively.

All participants showed significantly enhanced N100 activation for chirp compared to click conditions, with the responsible generators localized in the transverse temporal gyrus (TTG) (*t* = 3.920, *p* = 0.010; BA 41). A detailed within-group analysis revealed that the HC group showed a marginally significant enhancement of N100 activation for chirp compared to click conditions, localized in the TTG (*t* = 3.512, *p* = 0.075; BA 41). In the MDD group, patients showed increased but not significant N100 activation, with activity localized to the insula (*t* = 3.075, *p* = 0.302; BA 13).

Source localization contrasts of the P200 component between the MDD and HC groups revealed that MDD patients showed significantly reduced P200 activation in the middle temporal gyrus (MTG) (*t* = –4.072, *p* = 0.003; BA 39). As detailed in Table 3, the HC group showed a significant increase in P200 activation during the chirp stimulus (*t* = –4.022, *p* = 0.006; BA 39), whereas no significant voxels were observed during the click stimulus.

### 3.3. Spectrotemporal Characteristics of 40-Hz ASSRs in MDD

**Figure 3:**
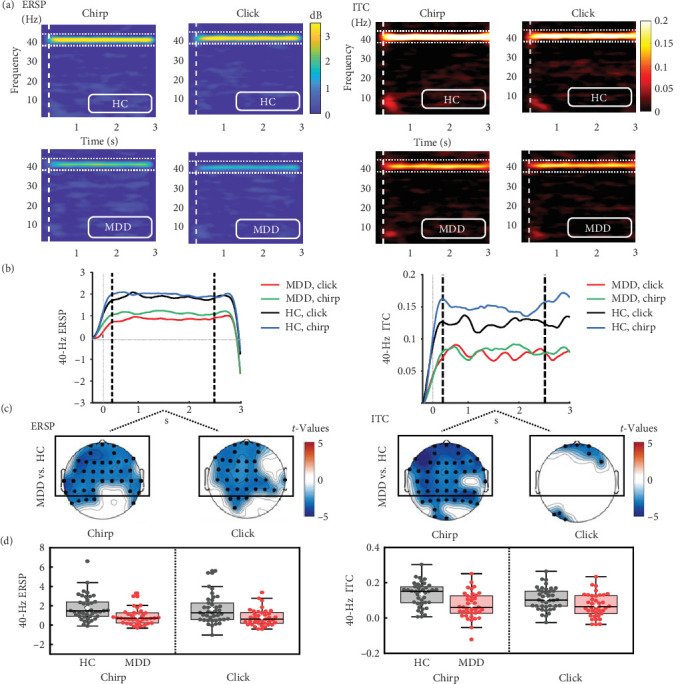
Main group differences in 40-Hz auditory entrainment responses. (a) The time–frequency plots of ERSP (left) and ITC (right) between the MDD and HC groups during chirp and click stimuli. Stimulus onset at time 0 and offset at 3000 ms. (b) The time course of 40-Hz ERSP (left) and ITC (right). The red and green curves represent the click-ASSR and the chirp-ASSR, respectively, in the MDD group; the black and blue curves represent the click-ASSR and the chirp-ASSR, respectively, in the HC group. (c) Topographies of 40-Hz ERSP and ITC (MDD vs. HC) with significantly different electrodes. (d) The box plots with mean (white cross) and median (solid line) of the average ERSP and ITC (average of the electrodes marked by black rectangles in (c)) were made separately for the HC (black dots) and MDD (red dots) groups. ERSP, event-related spectral perturbation; HC, healthy control; ITC, inter-trial phase coherence; MDD, major depressive disorder.

Compared to HC, MDD showed impaired gamma activity throughout the steady-state period (500–2500 ms poststimulus; Figure 3a,b). The significant group differences in 40-Hz entrainment power showed a more anterior distribution involving frontal, temporal, and central regions for both stimuli (*p* < 0.01; Figure 3c). Therefore, we examined the average ERSP in the anterior regions and a mixed model ANOVA revealed a significant main effect of Group (*F*_(1, 78)_ = 13.032, *p* = 0.001; Figure 3d), with a reduced 40-Hz power in MDD compared to HC for both stimuli. However, the interaction effect of Group × Stimulus and the main effect of Stimulus were not significant.

In addition, MDD presented a global attenuation in the 40-Hz ITC to the chirp stimulus, but a local attenuation to the click stimulus. Similarly, we examined the average ITC in the anterior regions, and a mixed model ANOVA revealed a significant main effect of Group (*F*_(1, 78)_ = 14.559, *p* = 0.0003) and a marginally significant interaction effect of Group × Stimulus (*F*_(1, 78)_ = 3.730, *p* = 0.057). The main effect of Stimulus was marginally significant (*F*_(1, 78)_ = 3.145, *p* = 0.080), suggesting that MDD showed a greater deficit in phase synchronization across trials during the chirp stimulus.

### 3.4. Connectivity Patterns of 40-Hz ASSRs in MDD

**Figure 4:**
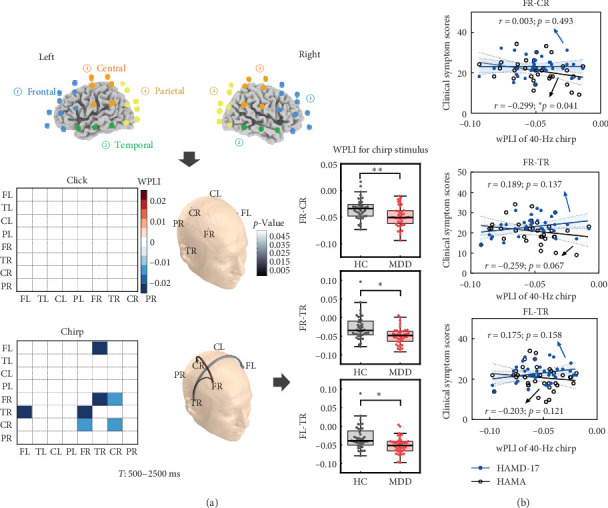
Connectivity in the 40 Hz auditory response between the MDD and HC groups. (a) Inter-regional connectivity between the two groups during click and chirp stimuli. The top row shows the eight defined ROIs including the left frontal (FL), left temporal (TL), left central (CL), left parietal (PL), right frontal (FR), right temporal (TR), right central (CR), and right parietal (PR) regions. Below are the corresponding heat maps and connectivity networks for all regions during click and chirp stimuli. (b) Correlations between significantly attenuated WPLI and clinical symptom scores during the chirp stimulus. Right frontocentral connectivity showed a significant association with patients' HAMD-17 and HAMA scores. Besides, connectivity between bilateral frontal and right temporal regions correlated significantly with HAMA scores. *⁣*^*∗*^*p* < 0.05; *⁣*^*∗∗*^*p* < 0.01. HC, healthy control; MDD, major depressive disorder; WPLI, weighted phase-lag-index.

We observed that patients with MDD exhibited significantly reduced connectivity compared to HCs during the chirp stimulus (Figure 4a), including connections between FR and TR (*p* = 0.008), between FR and CR (*p* = 0.017), and between FL and TR (*p* = 0.048), suggesting that MDD is characterized by impaired right cortical communication. Notably, the attenuated connectivity was specific to the chirp stimulus, as no significant group difference was found for the click stimulus. Furthermore, connectivity between the FR and CR regions was significantly negatively correlated with HAMA scores in MDD patients during the chirp stimulus (Spearman's *r* = –0.299, *p* = 0.041, Figure 4b). In addition, HAMA scores showed a marginally significant negative correlation with connectivity between the FR and TR regions (Spearman's *r* = –0.259, *p* = 0.067).

## 4. Discussion

By combining AEPs and ASSRs, we present a promising stimulation—the chirp stimulus—for assessing aberrant gamma oscillations in first-episode drug-naïve patients with MDD. Two main findings emerged from the current study. First, patients with MDD showed substantial alterations in gamma auditory responses, characterized by a prominent reduction in P200 activity, suppressed 40-Hz entrainment power and ITC in the anterior cortex, and insufficient functional connectivity in the right hemisphere, in contrast to HCs. Second, attenuated right frontocentral connectivity was strongly related to the severity of depression and anxiety symptoms. Notably, these findings were stimulus driven, that is, the abnormalities associated with clinical symptoms of MDD were found with the chirp stimulus, but not with the click stimulus.

As expected, our results provided direct evidence that patients with MDD have shown a prominent reduction in P200 amplitude, consistent with previous research suggesting early auditory processing dysfunction in MDD [60, 61]. Boutros et al. [62, 63] proposed a link between the P200 component and resource allocation in auditory processing. Subsequently, Ross and Fujioka [64] further elucidated that the P200 component signifies the successful completion of the process whereby the neural representation of an auditory object is established and can be subconsciously processed. The reduced P200 amplitude observed in MDD patients may indicate an effect on the formation and perception of auditory objects during auditory processing. In particular, the P200 component is considered to be an indirect indicator of central serotonin function [65]. Given that serotonergic dysfunction in the central nervous system is thought to be one of the major pathophysiological factors in MDD and that serotonin plays a mediatory role in the generation of the ASSR [25], this physiological basis underpins the investigation of the association between P200 and ASSR indices. It is worth noting that some studies have found increased P200 amplitudes in MDD [66, 67] possibly due to variations in stimulation modality or intensity. Whereas previous research has used high-intensity stimuli (70- and 80-dB SPL) [68], our study used low-intensity stimuli, providing a plausible explanation for the divergent findings. Specifically, we found that reduced P200 activity was localized in the MTG during the chirp stimulus. A meta-analysis supports this perspective, showing a reduction in MTG gray matter in first-episode MDD [69, 70]. Clearly, our results have shed additional light on the abnormal P200 patterns in first-episode MDD. Recent evidence from Parker et al. [71, 72] suggests that earlier reductions in evoked responses (e.g., P200) serve as a marker of severity in severe mental illness. However, the relationship between P200 activity and depressive symptoms remains to be elucidated. Future studies that expand the sample size to investigate the relationship between the P200 component and clinical symptoms of MDD during click and chirp stimuli, particularly with an emphasis on source-level analysis, will provide a promising avenue for research into the neurobiological basis of the aberrant auditory processing patterns observed in patients with MDD.

In our previous research, we implemented an ASSR paradigm using click stimulus that alternated between 40 and 60 Hz and found a reduction in right hemisphere 40-Hz ITC in patients with MDD [73]. Capitalizing on these findings, the current study focused on the 40-Hz stimuli and incorporated chirp signals to conduct an in-depth investigation, with the aim of uncovering the aberrant auditory response patterns in MDD. Our comparison of 40-Hz entrainment power in the auditory stimulus period and baseline revealed gamma activation in the limbic lobe. The limbic system receives either direct or indirect neural input from the auditory system [74]. Dysfunction of the limbic system may underlie depressive mood disorders [75]. In addition, our results showed that the 40-Hz entrainment abnormalities involve reduced ERSP and ITC in frontal, temporal, and central brain regions. The attenuated power and phase consistency of neural responses in MDD during a 40-Hz ASSR paradigm, possibly reflecting an inability to generate or maintain gamma activity. Specifically, with respect to the phase consistency of 40-Hz ASSRs, the marginally significant interaction effect of Group × Stimulus, together with the main effect of Stimulus, indicated that MDD exhibited a more severe deficit in phase synchronization across trials, particularly during the presentation of chirp stimuli. Furthermore, most previous studies have used relatively short (500–1000 ms) trains of 1-ms clicks [23], and analyzed 500-ms stimulus durations, whereas the ASSR requires at least 240 ms to develop [76]. Combined with longer stimulus durations (3000 ms), our stimulus presentation may have driven the abnormal cortical neural responses in MDD. Future studies with different stimulus conditions are needed to better understand these discrepancies.

Functional connectivity is emerging as a critical neurophysiological endophenotype of MDD, highlighting its importance in research. A recent resting-state EEG study showed that MDD patients have weaker functional connectivity between occipital and parietal regions of the right hemisphere in the 4–30 Hz range compared to the HC group [77]. While studies of gamma functional connectivity have generally not reported significant differences between MDD and HC groups. In their review of the literature, Miljevic et al. [78] suggested that gamma activity may be more pronounced during task-related processes, which may partially explain the variability in findings. In our study, we first observed that MDD disrupted the “gamma-modulated” functional interaction between frontal cortex and right temporal cortex during the chirp stimulus. Given that the right auditory cortex plays a central role in frequency discrimination [79], disturbances in MDD may reflect the absence of appropriate task-related neural facilitation. These findings are consistent with those of Zweerings et al. [80], who found disrupted frontotemporal connections in this disorder during an auditory mismatch task. In addition, EEG-derived functional connectivity is thought to be strongly influenced by white matter myelinated corticocortical axons [81]. Studies have shown that in the absence of white matter, functional connectivity is significantly reduced for high-frequency bands compared to low-frequency bands [81, 82]. Therefore, it is imperative for future research to investigate the interplay between white matter structure and gamma functional connectivity in MDD, and in particular to elucidate how white matter provides structural support for the coupling of gamma activity. In addition, functional connectivity between the right frontal and central cortex was significantly associated with patients' HAMA scores. Roh et al. [14] reported that frontocentral gamma oscillations are associated with symptoms of inattention, which in turn appear to be related to subjective symptom severity. Future studies using the inattention subscale of the Adult ADHD Scale or an integrative approach in conjunction with other methods are needed to clarify the role of gamma synchrony and attention deficits in MDD.

It should be noted that when evaluating 40-Hz ASSRs, the effect in the gamma band should be interpreted with caution due to the risk of contamination of the EEG signal by, for example, muscle artifacts. For example, Bidelman et al. [83] pointed out that the use of a mastoid reference electrode would promote extraneous recording of postauricular muscle (PAM) artifacts. The PAM is part of a vestigial startle reflex, responsible for retracting the pinna to protect hearing, and can produce large bilateral contractions (>100 µV) [84]. In the present study, PAM muscle artifacts were minimized by using low-intensity stimuli, averaging references, and discarding epochs with voltages greater than 100 µV. The observed 40-Hz ASSRs were widespread and generally present in the central regions of the recording electrodes, where contamination by, for example, muscle artifacts is expected to be low. Therefore, the findings within the 40-Hz ASSRs appear to be robust, but it is still emphasized that any effects observed in high-frequency oscillations should be interpreted with caution.

Finally, our findings represent a novel application of chirp stimuli to the analysis of auditory responses in MDD. Artieda et al. [85] proposed that chirp-modulated tones provide a more global view of brain activity. In the present study, we found larger N100 activation with the chirp stimulus compared to the click stimulus, which was source-localized to the transverse temporal gyrus (TTG) or insula. The TTG, as an important structure of the auditory cortex, is closely related to sound frequency. Several fMRI studies have suggested that the medial half of the TTG contains a tonotopic gradient, with more medial aspects processing high-frequency sound information and more lateral aspects processing low-frequency sound information [86, 87], which is consistent with the formation principle of chirp signals [40]. The TTG plays an important role in the primary auditory center responsible for the perception and processing of external auditory information [88]. In addition, the N100 is considered to be an index of early information registration of stimuli (i.e., attention) [32], suggesting that the chirp stimulus may elicit greater early attention in the primary auditory cortex compared to the click stimulus.

**Figure 5:**
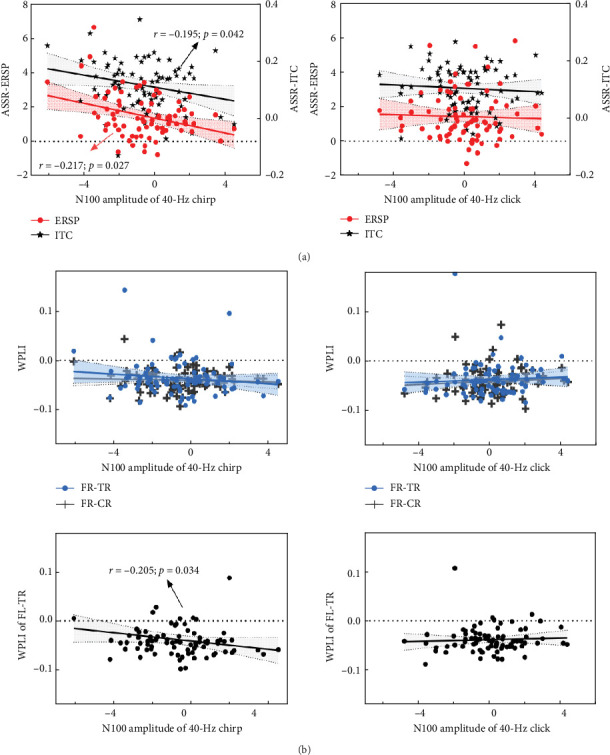
Correlations between N100 amplitude and 40-Hz auditory responses at FCZ, CZ, and CPZ electrode sites. (a) Correlations between N100 amplitude and 40 Hz ERSP (red dots) and ITC (black asterisks) during chirp (left) and click (right) stimuli. During the chirp stimulus, the N100 amplitude was significantly negatively correlated with both 40-Hz ERSP and ITC. (b) Correlations between N100 amplitude and 40-Hz WPLI for chirp (left) and click (right) stimuli. No significant correlations were found for intrahemispheric connections (top), as shown by the WPLI between FR and CR (black plus signs), and the WPLI between FR and TR (blue dots). For interhemispheric connections (bottom), the N100 amplitude was significantly negatively correlated with the WPLI between FL and TR during the chirp stimulus. No significant correlation was found during the click stimulus. ITC, inter-trial phase coherence; WPLI, weighted phase-lag-index.

Interestingly, more pronounced abnormalities of the 40-Hz entrainment responses were also observed during the chirp stimulus in this study. Galambos, Makeig, and Talmachoff [41] suggested that the 40-Hz ASSR is essentially related to the N100 component, and several reports [89] have found empirical evidence to support this, while others have not [90]. More recently, a growing body of evidence suggests that the N100 component and ASSRs may not represent the same underlying neural substrate but are likely to share some overlap [91]. Considering the basic findings of existing research, we speculate that the N100 component may exert some influence on ASSRs in MDD, even in the absence of detectable abnormalities. To investigate the contribution of the N100 to the 40-Hz entrainment variables (ERSP, ITC, and WPLI), Spearman's correlations were performed in this study. During the chirp stimulus, the average N100 amplitude was significantly negatively correlated with both 40-Hz ERSP (Spearman's *r* = –0.217, *p* = 0.027) and ITC (Spearman's *r* = –0.195, *p* = 0.042) in the FCZ, CZ, and CPZ electrodes (Figure 5a). Meanwhile, the N100 amplitude was significantly negatively correlated with the 40-Hz WPLI between FL and TR (Spearman's *r* = –0.205, *p* = 0.034; Figure 5b). However, no significant correlations were found during the click stimulus (ERSP: *p* = 0.206; ITC: *p* = 0.203; WPLI: all *p* > 0.2). The prominent N100 component, which is a negative deflection, indicates impaired early auditory processing when it appears with an increased amplitude, as this corresponds to a reduced absolute value. Thus, we speculate that the ability of the cerebral cortex to generate and transmit gamma oscillations during the chirp stimulus may depend on its ability to generate the N100 and integrate early auditory information. Moreover, these findings may be due to the superior signal-to-noise ratio enjoyed by transient AEP and ASSR measures. Future research is needed to elucidate the physiological basis of the 40-Hz ASSR during chirp stimulation, and then to characterize the relationship between ASSR and AEP deficits in patients with MDD.

## 5. Conclusions

Our data provide initial evidence for deficits in AEPs and 40-Hz ASSRs in first-episode drug-naïve patients with MDD using a chirp stimulus. Patients with MDD showed a prominent reduction in P200 activity, suppressed 40-Hz entrainment power and ITC in the frontocentral cortex, and insufficient functional connectivity in the right hemisphere compared to HCs. Importantly, attenuated right frontocentral connectivity was strongly related to anxiety severity in MDD. Notably, these findings were stimulus driven, that is, the abnormalities associated with clinical symptoms of MDD were found only with the chirp stimulus but not with the click stimulus. Our findings highlight that symptom-related right frontocentral connectivity elicited by the chirp stimulus may be a promising signature for predicting depression severity and support the potential usefulness of the chirp stimulus as a novel approach to reveal the specific neurophysiological signatures of MDD.

## Figures and Tables

**Table 1:** Demographic and clinical information.

Characteristics	HC	MDD	Statistics	*p*-Value
Sample size	41	40	—	—
Sex, male^a^	18	12	*χ* ^2^_(1)_ = 1.678	0.252
Age, year^b^	33.12 ± 7.47	31.53 ± 9.14	*Z* = −1.296	0.195
Education, years^b^	14.27 ± 3.45	14.93 ± 2.65	*Z* = −0.672	0.501
Handedness, right^a^	40	38	*χ* ^2^_(1)_ = 0.372	0.616
Ethnicity, Han/others^a^	38/3	37/3	*χ* ^2^_(1)_ = 0.001	1.000
HAMD-17 scores^c^	1.21 ± 1.34	23.35 ± 5.10	*t*_(79)_ = 26.234	<0.001
HAMA scores^c^	1.23 ± 1.27	22.10 ± 7.34	*t*_(79)_ = 17.507	<0.001

*Note:* Results are expressed as mean ± standard deviation (SD).

^a^Chi-square test.

^b^Mann–Whitney *U*-test.

^c^Independent samples *t*-test.

**Table 2:** Source localization contrasts for the N100 component between chirp and click conditions, separately for HC and MDD groups.

Lobe	Best match (mm)	Region	Brodmann areas	*t*-Score	MNI (*XYZ*)
All participants					
Temporal	0	Transverse temporal gyrus	41	3.92	−35, –35, 10
	7	Insula	13	3.73	−30, –30, 15

HC group					
Temporal	0	Transverse temporal gyrus	41	3.51	−35, –35, 10
	7	Insula	13	3.25	−35, –40, 20

MDD group					
Temporal	0	Insula	13	3.08	−40, –25, 20
	7	Insula	41	2.66	−45, –25, 15

*Note:* The critical *t*-scores for all participants, the HC group, and the MDD group are 3.57, 3.63, and 3.59, respectively, corresponding to *p* = 0.05.

Abbreviations: HC, healthy control; MDD, major depressive disorder.

**Table 3:** Source localization contrasts for the P200 component between MDD and HC groups, separately for chirp and click stimuli.

Lobe	Best match (mm)	Region	Brodmann areas	*t*-Score	MNI (*XYZ*)
All stimuli					
Temporal	0	Middle temporal gyrus	39	−4.07	−40, –65, 20
	7			−4.01	

Chirp stimulus					
Temporal	0	Middle temporal gyrus	39	−4.02	−40, –65, 20
	7			−3.93	−45, –75, 25
Click stimulus					

Limbic lobe	0	Anterior cIngulate	33	−2.91	0, 20, 20
	7			−2.90	5, 20, 20

*Note:* Critical *t*-scores for all stimuli, chirp stimulus, and click stimulus are 3.42, 3.45, and 3.33, respectively, corresponding to *p* = 0.05.

Abbreviations: HC, healthy control; MDD, major depressive disorder.

## Data Availability

Data are available from the first or corresponding author upon reasonable request. The data are not publicly available as they contain private information about the study participants.
